# EP15 A self-limiting symmetrical polyarthritis following COVID-19 infection

**DOI:** 10.1093/rap/rkaa052.014

**Published:** 2020-11-03

**Authors:** Mark Gibson, Karan Sampat, Gerald Coakley

**Affiliations:** r1 Lewisham and Greenwich NHS Trust, London, United Kingdom; r2 Darenth Valley Hospital, London, United Kingdom

## Abstract

**Case report - Introduction:**

We describe an acute onset self-limiting seronegative non-destructive symmetrical polyarthritis five weeks after laboratory confirmed COVID-19 infection.

**Case report - Case description:**

A 37-year-old male hospital doctor of presented to the Early Inflammatory Arthritis clinic with a four-week history of acute onset joint pain, swelling and early morning stiffness in excess of two hours. The symptoms began at the left ankle with Achilles’ tendonitis but progressed over the following 72 hours to a symmetrical polyarthritis affecting the wrists, proximal interphalangeal joints, shoulders, elbows, and knees.

Approximately five weeks prior to the onset of his joint symptoms he had laboratory confirmed SARS-CoV-2 infection with six days of fever, non-productive cough, and fatigue. He did not require hospitalisation.

His past medical history was significant for biopsy proven non-alcoholic fatty liver disease. There was no prior history of inflammatory arthritis and no personal or family history of skin psoriasis, inflammatory bowel disease or uveitis. There was no preceding genitourinary or gastrointestinal upset. His family history was significant for a sister with seronegative rheumatoid arthritis for which she was taking sulfasalazine.

Examination revealed a normal BMI, synovitis at the wrists and proximal interphalangeal joints without evidence of joint effusion in the large joints. Blood tests revealed elevations in the ESR (83 mm/hour, reference range 0-10 mm/hour) and CRP (25mg/dL, reference range <5mg/dL). Serology was negative for the rheumatoid factor, anti-CCP antibodies, antinuclear antibodies, and an extractable nuclear antigen panel. Radiographs of the affected joints were unremarkable. Serological testing was positive for anti-SARS-CoV-2 IgG antibodies.

He was started on oral Prednisolone 20mg daily and an NSAID with good symptomatic response and normalisation of his ESR (5mm/hour) and CRP (<1mg/dL). The course of prednisolone was tapered over a 6-week period and he is still in steroid free remission with normal inflammatory markers at follow up. The patient was given a diagnosis of a post-viral reactive arthritis which was attributed to the preceding COVID-19 illness.

**Case report - Discussion:**

Post infectious inflammatory arthritis has been described with many viral infections including: hepatitis virus, parvovirus B19, enterovirus, rubella, alphavirus (including Chikungunya), flavivirus (including Zika), herpes viruses (including Epstein-Barr virus), varicella, cytomegalovirus and human immunodeficiency virus (HIV). Interestingly, viral arthritis has not been reported in influenza and human coronaviruses (including SARS and MERS). Arthralgia was reported in 14.9% of laboratory confirmed COVID-19 cases in China during the early phases of the pandemic but inflammatory arthritis was not well described.

The clinical course of the inflammatory arthritis in this case was self-limiting with enthesitis and synovitis resolving within six weeks of onset with the mainstay of treatment being symptomatic relief in the form of non-steroidal anti-inflammatory drugs and corticosteroids.

Patient perspective: When I woke up that Tuesday morning with severe joint pains and stiffness, I knew something was not right. It was not like anything I have felt before in terms of my joints, having had sports injuries in the past. It was to the point where I was even struggling to go from sitting to standing. Without Prednisolone, I feel as if I would not have been able to work and may even have been house bound. I was relieved that this inflammatory arthritis did respond to Prednisolone. After six weeks of taking Prednisolone, the condition seemed to settle.

**Case report - Key learning points:**

A self-limiting episode of inflammatory arthritis may occur following COVID-19 infection.

